# Phytochemical Profiles and Antimicrobial Activity of *Alnus glutinosa* (L.) Gaertn. Leaves Growing in Kazakhstan

**DOI:** 10.3390/molecules31071189

**Published:** 2026-04-03

**Authors:** Aliya Bazargaliyeva, Janar Jenis, Yergazy Shybyray, Gulnur Admanova, Zhaidargul Kuanbay, Samal Duzelbayeva, Balzat Sarimbayeva, Gulzhakhan Kaisagaliyeva, Bagdagul Alzhanova, Rima Kozhagaliyeva

**Affiliations:** 1 Department of Biology, Natural Sciences Faculty, K.Zhubanov Aktobe Regional University, 34 Moldagulova Avenue, Aktobe 030012, Kazakhstan; gadmanova@zhubanov.edu.kz (G.A.); zkuanbay@zhubanov.edu.kz (Z.K.); bsarimbayeva@zhubanov.edu.kz (B.S.); 2 Research Center for Medicinal Plants, Al-Farabi Kazakh National University, Al-Farabi Ave. 71, Almaty 050040, Kazakhstan; janarjenis@kaznu.kz (J.J.); erhazi88@mail.ru (Y.S.); 3 Department of Chemistry and Food Technology, Natural Sciences Faculty, K.Zhubanov Aktobe Regional University, 34 Moldagulova Avenue, Aktobe 030012, Kazakhstan; sduzelbayeva@zhubanov.edu.kz; 4 Department of Biology and Ecology, Makhambet Utemisov West Kazakhstan University, 162 N. Nazarbayev Avenue, Uralsk 090000, Kazakhstan; zapkazu@wku.edu.kz (G.K.); aljanb@mail.ru (B.A.); 5 Educational Programs for the Training of Teacher Biology, Geography, Chemistry, Natural Geography Faculty, Makhambet Utemisov West Kazakhstan University, 162 N. Nazarbayev Avenue, Uralsk 090000, Kazakhstan; rabdrakhmanova_7@bk.ru

**Keywords:** *Alnus glutinosa* (L.) Gaertn., antimicrobial activity, mineral composition, polyphenolic compounds, HPLC, GC-MS

## Abstract

*Alnus glutinosa* (L.) Gaertn. has extensive use in traditional medicine and diverse biological activities due to its rich phytochemical profile. In this study, firstly, the physicochemical characteristics of the plant material were evaluated, revealing a high content of extractive substances (17.684%), followed by ash (6.740%) and moisture (5.000%). Among the bioactive constituents, tannins were the most abundant (7.439%). Analysis of macroelements in the plant ash showed K (11.4330 mg/g) as the predominant element, followed by Mg (97.13 mg/g), Ca (75.30 mg/g), and Na (72.41 mg/g). Trace element analysis indicated Fe (1.2266 mg/g) as the most abundant microelement, with Zn (0.8870 mg/g) and Mn (0.8141 mg/g) present in comparable amounts. Gas chromatography–mass spectrometry (GC-MS) analysis of the ethanolic leaf extract characterized volatile and semi-volatile constituents of 43 phytochemical components, where vitamin E was the predominant compound (20.52%), followed by phytol (12.46%) and squalene (10.29%). Further high-performance liquid chromatography (HPLC) analysis confirmed the presumed presence of naringin (56.421 mg/L), followed by epicatechin (15.123 mg/L), catechin (12.485 mg/L), and phloridzin (11.800 mg/L), while gallic acid was detected at a comparatively lower concentration (0.402 mg/L). The antimicrobial activity of the aqueous leaf extract was evaluated against typical Gram-positive and Gram-negative pathogens, including *Staphylococcus aureus*, *Salmonella abony*, *Escherichia coli*, and *Klebsiella pneumoniae*. To evaluate the effect of compositional changes on antimicrobial activity, the fermented and non-fermented formulations of *A. glutinosa* leaf extracts were prepared. These results demonstrate measurable antibacterial effects, thereby confirming the ethnopharmacological significance of *A. glutinosa* and highlighting its potential as a source of natural antimicrobial agents for further pharmacological development.

## 1. Introduction

*Alnus glutinosa* (L.) Gaertn. is a member of the *Betulaceae* family, which is also called “common alder, european alder, black or sticky alder” in traditional medicine. More than 40 species in this genus can be found along the shores of lakes, rivers and streams of Europe, Western Siberia, Southeastern Asia, Africa and North America. In Kazakhstan, it is distributed in Karkarala, Ereimentau, Ilek, Oral, Aktobe, Turgay and Bayanaul regions; however, its phytochemical composition and biological activity remain poorly understood in populations growing in Kazakhstan [1]. *A. glutinosa* is a sacred plant that is resistant to growing in various conditions, easily absorbing nutrients from the soil and restoring its quality [2,3]. It is sold on the market as a dietary supplement that helps reduce the risk of various chronic dermatological conditions. Common alder barks have been used in traditional medicine as an astringent, cathartic, anti-lice agent, febrifuge, anti-swelling agent, anti-emetic, anti-sore throat agent, skin problem-resolving agent, hemostatic, anti-rheumatic agent, tonic, anti-pharyngitis agent and bitter [4,5]. In Turkish folk medicine, *A. glutinosa* leaves are used to heal wounds, rheumatism and cuts [6]. The fruits of *A. glutinosa* are used to treat acute and chronic enteritis, colitis, dysentery, and cold, and are used as a mouthwash for the oral cavity [7].

According to the previous studies, *A. glutinosa* has several biological activities including antimicrobial, antioxidant [8,9], anti-inflammatory, hepatoprotective, antibacterial, anti-fungal, antitumor [10], anti-angiogenic [11] and anti-toxoplasma gondii [12]. Chemical composition studies have shown the presence of various compounds in *A. glutinosa*, including phenols, sterols, tannins, flavonoids, triterpenes, phenolcarboxylic acids, phenolic acids, ellagitannins, flavonoid glycosides, ellagic acid derivatives, and diarylheptanoids. The recent literature has increasingly emphasized the role of these compounds in disrupting microbial cell walls, inhibiting enzymatic systems, and reducing oxidative stress in pathogenic microorganisms, confirming their pharmacological significance [10,13]. Diarylheptanoids (1,7-diphenylheptanes) are among the main phenolic compounds of the genus *Alnus* [14]. Approximately 20 such compounds have been isolated from *A. glutinosa*, possessing antibacterial, antioxidant, chemoprotective, antiviral, and antitumor activity [15,16].

The species of the genus *Alnus* are traditionally used in medicine and are characterized by a rich phytochemical composition and a broad spectrum of biological activity. The aim of this study is to comprehensively assess the biological potential of *A. glutinosa,* which grows in Kazakhstan, and to investigate the active compounds in the plant material. For this purpose, physicochemical parameters (moisture, ash content, extractive substances, macro- and microelement composition) were determined, and the phytochemical profiling of the ethanolic extract was performed using HPLC and GC-MS, and the antimicrobial activity of the aqueous extract was assessed.

## 2. Results and Discussion

### 2.1. Quantitative and Qualitative Analyses

The quantitative and qualitative analyses of moisture, ash content, extractive substances and bioactive constituents (polysaccharides, flavonoids, organic acids, coumarins, alkaloids, tannins) in the leaves of *A. glutinosa* plants were performed, and their results are shown in Table 1. Extractive substances show the highest content (17.684%), followed by ash (6.740%) and moisture content (5.000%), while among the bioactive compounds, tannins (7.439%) were found to be the most abundant components. Physicochemical parameters such as moisture and ash content are essential for standardization, while extractive values—particularly in ethanol—correlate with the higher recovery of bioactive constituents, as shown by the Soxhlet extraction yielding superior antioxidant and photoprotective activity in cosmetic formulations [17]. Phytochemical investigations confirm the presence of major classes including flavonoids, polyphenols, tannins, and organic acids in *A. glutinosa* leaves, which are consistent with the compounds identified in the present study [18]. Previous reports also suggest that diarylheptanoids are characteristic of the genus; however, they were not quantified in our analysis. Chromatography–mass spectrometry analysis has identified multiple organic acids in leaves, with oxidal acid reported as the predominant component [19], and it is a group in the organic acid profile observed in our results. Collectively, these findings support the metabolic diversity and biological potential of *A. glutinosa* leaves, reinforcing their relevance for pharmacological and cosmeceutical applications.

### 2.2. Mineral Composition Analysis

Macro- and microelements are essential nutrients required for human health in varying amounts for energy, structural purposes, enzyme activity and cell processes. The average amounts of mineral macro- (K, Mg, Ca and Na) and microelements (Cu, Fe, Zn, Ni, Mn and Pb) in the ash of dry leaves of *A. glutinosa* were recorded using atomic absorption spectroscopy, as shown in Table 2. Among the macroelements detected in plant ash, K (11.4330 mg/g) showed the highest amount followed by Mg (97.13 mg/g), Ca (75.30 mg/g) and Na (72.41 mg/g). Among the trace elements, Fe (1.2266 mg/g) is the most abundant microelement, Zn (0.8870 mg/g) and Mn (0.8141 mg/g) are in approximately equal amounts, Cu (0.4121 mg/g) and Ni (0.2167 mg/g) are relatively low compared to other trace elements, Cd (0.0170 mg/g) is detected in the lowest amount, and Pb is not detected at all.

Analysis of the mineral composition of *A. glutinosa* demonstrates its high adaptive capacity in both natural and contaminated environments [2]. The seasonal monitoring of leaf elemental contents revealed the adequate levels of N, S, Ca, and Mg, while P and K were often low; the elevated concentrations of Mn, Zn, and Al were also noted, reflecting soil chemistry and stressful environmental conditions [20]. In metal-contaminated soils, both *A. glutinosa* and *A. incana* preferentially accumulated Cu, Pb, Zn, and Cd in roots and nodules, with limited translocation to leaves, suggesting a defense exclusion mechanism maintaining leaf metal concentrations within normal physiological ranges [21]. Nutrient ratios (P:K:Ca:Mg) and nitrogen use efficiency varied depending on soil conditions, with high nitrogen use efficiency observed on reclaimed mining sites despite low initial phosphorus content [22]. Taken together, these data confirm that *Alnus* species exhibit the efficient regulation of macro- and micronutrients, tolerance to heavy metals, and ecological suitability for soil restoration and phytostabilization.

In contrast to previously published ecological studies of *A. glutinosa*, which focused on seasonal variability and heavy metal accumulation under environmental stress, the present mineral analysis of leaf ash provides a more direct quantitative characterization of macro- and micronutrients. The predominance of K (11.4330 mg/g), followed by Mg, Ca, and Na, confirms that potassium is the major macronutrient in *A. glutinosa* leaves, consistent with its physiological role in osmotic regulation and metabolic activity. Among the micronutrients, Fe was the most abundant, while Zn and Mn were present in comparable amounts, confirming the earlier observations of elevated Mn and Zn levels in alder foliage. Importantly, the absence of detectable Pb and very low Cd concentrations indicate the minimal accumulation of toxic metals in this material, which contrasts with reports from contaminated soils, where root sequestration limits migration to leaves. Overall, these results highlight both the richness of nutrient minerals in *A. glutinosa* leaves and their ability to regulate potentially harmful elements.

### 2.3. Determination of Phytochemicals by GC-MS

**Figure 1:**
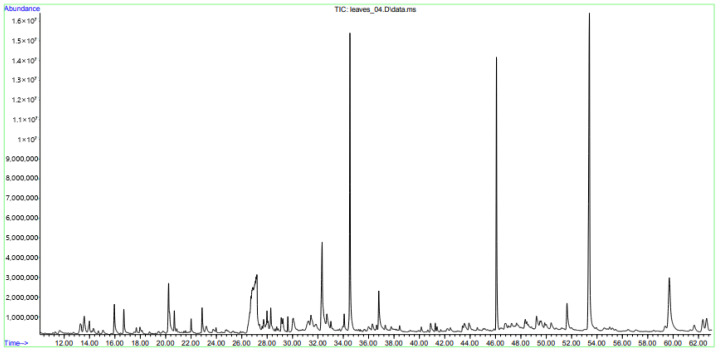
GC-MS chromatogram of ethanolic extract from *A. glutinosa* leaves.

**Figure 2:**
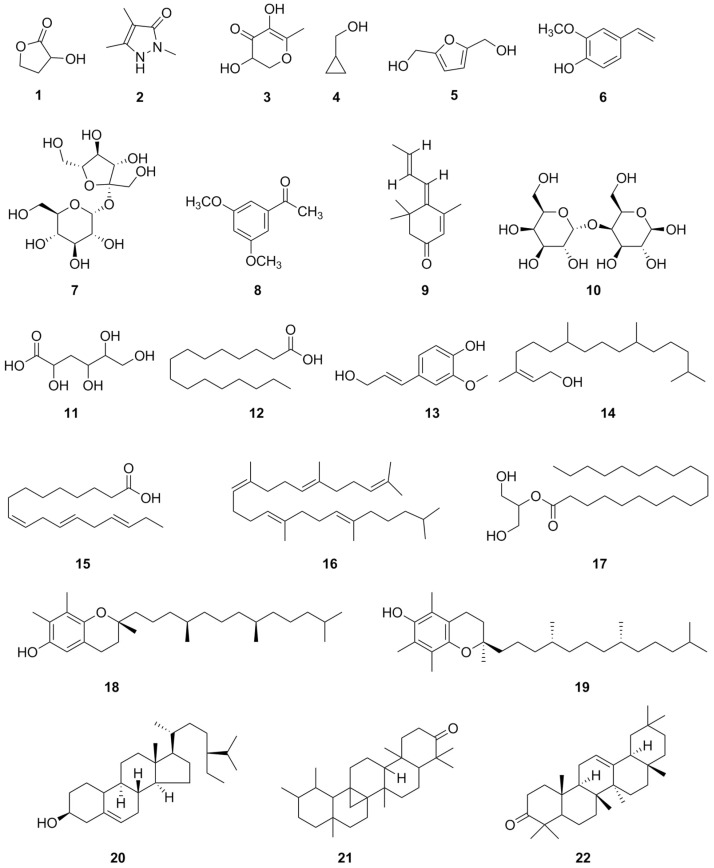
The structures of compounds identified in *A. glutinosa* leaves using GC-MS, with corresponding names provided in Table 3.

GC-MS is an essential tool for pharmaceutical and phytochemical research, enabling the accurate characterization of volatile and semi-volatile constituents and the qualitative measurement of these compounds. It supports quality control, compound characterization, and bioactive molecule isolation, and plays a crucial role in solving industrial R&D challenges and advancing new drug development. The GC-MS analysis of the ethanolic extract of *A. glutinosa* leaves found 43 bioactive compounds (Figure 1) with significant biological and therapeutic properties. The most abundant compound was vitamin E (20.52%), followed by the notable levels of phytol (12.46%) and squalene (10.29%). Hexadecanoic acid (7.04%), γ-Sitosterol (7.48%), sucrose (5.42%), 5-Hydroxymethylfurfural (4.19%), and 9,12,15-Octadecatrienoic acid, (*Z*,*Z*,*Z*)- (2,89%) appeared in moderate amounts, while all other compounds were present at concentrations below 2%. Compounds with a concentration exceeding 1% are classified as the main compounds. The chemical structures of several major compounds detected in *A. glutinosa* leaves are presented in Figure 2. The retention time (RT), name, molecular formula (MF), molecular weight (MW) structure, and content of these compounds are presented in Table 3.

GC-MS has been widely used in the phytochemical studies of *Alnus* species, demonstrating its reliability for profiling volatile and semi-volatile components in various plant organs and using a variety of extraction methods. In *A. glutinosa*, GC-MS analysis after supercritical CO_2_ extraction identified 11 pentacyclic triterpenes and β-sitosterol, highlighting the method’s suitability for detecting lipophilic bioactive metabolites such as betulin, betulinic acid, and lupeol [23]. The comparative GC-MS studies of *A. subcordata* and *A. glutinosa* wood revealed remarkable chemical diversity (99 and 127 compounds, respectively), with species-specific dominant components and 12 common components, confirming chemotaxonomic differentiation within the genus [24].

Similarly, the GC/MS profiling of *A. cremastogyne* pod extracts identified up to 58 compounds depending on solvent polarity, including phytol, germacrene D, lupeol, and β-sitosterol, which correlated with their potent antioxidant and UV-protective activity [25]. In environmental monitoring studies, GC/MS was also effective for quantifying xenobiotic accumulation (e.g., HCH isomers) in *A. glutinosa*, demonstrating its applicability not only for phytochemical screening but also for environmental risk assessments [26]. Furthermore, the GC/MS/FID analysis of *A. incana* and *A. nitida* extracts identified terpenoids and fatty acids associated with cytotoxic and anti-inflammatory effects, linking chemical composition to biological activity [27,28,29,30,31].

Thus, the predominance of phytol, sterol, fatty acid, and terpenoid compounds in *A. glutinosa* is consistent with that reported in the previous studies of *Alnus* species, confirming the consistency of lipophilic metabolite profiles within the genus. The present study revealed that *A. glutinosa* leaves are rich in bioactive compounds and essential oils, exhibiting strong antioxidant, nutritional, and skin-protective properties. These constituents enhance oxidative stability, sensory quality, and functional performance, highlighting the promising potential of *A. glutinosa* leaves for applications in functional food products as well as pharmaceutical, biotechnological, and cosmetic industries.

### 2.4. Analysis of Selected Polyphenolic Compounds by Means of HPLC

**Figure 3:**
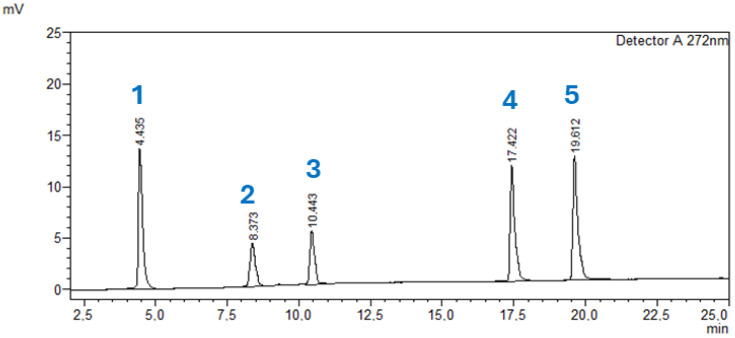
HPLC chromatogram of selected standards of phenolic compounds: gallic acid (**1**), catechin (**2**), epicatechin (**3**), naringin (**4**) and phloridzin (**5**).

The phytochemical composition of the *A. glutinosa* plant extract was analyzed using HPLC, with acetonitrile (1% acetic acid) in water serving as the mobile phase. Gallic acid, catechin, epicatechin, naringin, and phloridzin were used as reference standards for compound quantification. The HPLC chromatograms of these standards are shown in Figure 3. The retention times, peak areas, peak heights, and concentrations of each standard compound are presented in Table 4.

**Figure 4:**
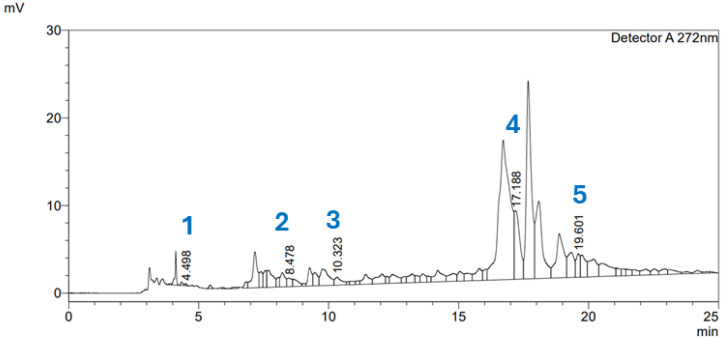
The HPLC spectra of the standard compounds gallic acid (**1**), catechin (**2**), epicatechin (**3**), naringin (**4**) and phloridzin (**5**) in the *A. glutinosa* extract.

The HPLC analysis confirmed the presumed presence of several phenolic and flavonoid compounds in the *A. glutinosa* extract. Naringin (**4**) was identified as the predominant compound, with a concentration of 56.421 mg/L, followed by epicatechin (**3**, 15.123 mg/L), catechin (**2**, 12.485 mg/L), and phloridzin (**5**, 11.8 mg/L). Gallic acid (**1**) was detected in a lower concentration (0.402 mg/L). These findings indicate that the extract is particularly rich in flavonoid constituents. The qualitative and quantitative results of the HPLC analysis are summarized in Figure 4 and Table 5.

The research findings on *A. glutinosa* (black alder) demonstrate a strong convergence between its long-established ethnomedicinal applications and contemporary pharmacological evidence. Traditionally used across Europe and parts of Asia to treat inflammatory conditions, skin disorders, infections, and gastrointestinal ailments, the bark, leaves, and cones of *A. glutinosa* exhibit broad therapeutic versatility. External applications for wound healing, burns, eczema, and ulcers are consistent with its astringent and anti-inflammatory properties, while internal uses for fever, respiratory complaints, rheumatic pain, and digestive disorders further emphasize its wide medicinal scope [18,32].

These traditional uses are strongly supported by the present findings, which identified several bioactive phenolic compounds in *A. glutinosa* extracts, including naringin (**4**), phloridzin (**5**), epicatechin (**3**), catechin (**2**), and gallic acid (**1**). These compounds are well known for their potent antioxidant and anti-inflammatory activities, providing a mechanistic basis for the plant’s effectiveness in managing inflammation and oxidative stress-related conditions [33,34]. In addition, their documented cardioprotective, antidiabetic, neuroprotective, antimicrobial, and anticancer properties suggest a broader therapeutic potential [35,36]. The ability of these phenolics to enhance endothelial function, regulate lipid metabolism, and improve insulin sensitivity further supports the traditional and contemporary use of *A. glutinosa* as a valuable health-promoting medicinal plant.

Overall, the presumed presence of these bioactive constituents provides a scientific basis for the ethnomedicinal applications of *A. glutinosa* and highlights its potential as a valuable source of natural compounds for therapeutic and preventive health applications.

### 2.5. Antimicrobial Activity

The development of fermentation-based formulations was driven by the goal of enhancing the bioavailability and transformation of phytochemicals present in *A. glutinosa* leaves. Fermentation is known to promote the release of bound phenolic compounds and the formation of low-molecular-weight metabolites, including organic acids, which may contribute to antimicrobial activity through pH reduction and membrane disruption mechanisms [37]. The inclusion of humic and fulvic acids was based on their known biological properties, including metal chelation, the modulation of microbial cell permeability, and the potential synergistic interactions with plant-derived compounds [38]. These substances may facilitate the transport of bioactive molecules across bacterial membranes and enhance overall antimicrobial efficacy. Furthermore, the acidic environment (pH ≈ 3) created during fermentation may have an additional inhibitory effect on microbial growth, particularly Gram-negative bacteria, by disrupting cellular homeostasis [39]. Similar approaches combining plant extracts with fermentation or organic acids to enhance antimicrobial activity have been reported, supporting the feasibility of the proposed formulation strategy [40].

To enhance the release and transformation of bioactive phytochemicals, as well as to generate organic acid metabolites that potentially promote antimicrobial activity, an aqueous extract (Sample **1**) of *A. glutinosa* was fermented (Sample **2**). The resulting acidic environment (pH ≈ 3), combined with humic (Sample **3**) and fulvic acids (Sample **4**), may exert an additive inhibitory effect on bacterial strains. The antimicrobial activity of four samples (**1**, **2**, **3**, and **4**) prepared from the aqueous extract of *A. glutinosa* leaves was evaluated against selected Gram-positive (*Staphylococcus aureus* ATCC 6538) and Gram-negative (*Salmonella abony* NTCC 6017, *Escherichia coli* NTCC 8439, *Klebsiella pneumoniae* ATCC 700603) bacterial strains. Activity was quantified by measuring the diameter of growth inhibition zones (mm) surrounding the wells, with all experiments performed in triplicate to ensure statistical reliability. According to the established criteria, inhibition zones less than 10 mm indicated weak or no antibacterial activity, approximately 10 mm indicated moderate sensitivity, and zones greater than 10 mm indicated high sensitivity.

All original samples and their 1:1 dilutions, with the exception of Sample **4**, demonstrated moderate antimicrobial activity against both Gram-positive and Gram-negative bacteria, as indicated by the inhibition zone diameters of approximately 10 mm. Sample **4** displayed a distinct response, as its 1:1 dilution exhibited a bacteriostatic effect, characterized by the inhibition of bacterial growth without any loss of cell viability.

Notably, the stock solution of Sample **1** (21 mm), consisting of an aqueous *A. glutinosa* leaf extract in 96% ethanol (2:1), showed pronounced antibacterial activity against *S. aureus*. Similarly, the stock solution of Sample **3** (20 mm), containing fermented *A. glutinosa* extract with acetic acid probiotics and humic acids (pH 3), demonstrated specific activity against *E. coli* NTCC 8439. These findings suggest selective antibacterial efficacy depending on the formulation and bacterial strain.

Overall, the results indicate that *A. glutinosa* leaf-derived preparations, particularly Samples **1** and **2**, possess promising antibacterial potential against clinically relevant bacterial pathogens. These findings support the potential therapeutic application of *A. glutinosa*-based extracts as natural antibacterial agents and warrant further investigation into their mechanisms of action and clinical efficacy. The results of the antimicrobial assays are shown in Table 6.

## 3. Materials and Methods

### 3.1. Plant Material

*A. glutinosa* leaves were collected from Karagash rural district, Aktobe region, Kazakhstan, on 20–24 September 2024. The collected leaves were air-dried in the shade and ground into small pieces and stored in the Department of Biology, Natural Sciences Faculty, Aktobe Regional University, named after Kudaibergen Zhubanov, Aktobe, Kazakhstan. The voucher specimen of the plant (KAZNU-20240925) was deposited at The Research Center for Medicinal Plants, Al-Farabi Kazakh National University, Almaty, Kazakhstan.

### 3.2. Reagents and Equipment

The mineral composition of the plant was determined using a Shimadzu 6200 series spectrometer (Shimadzu Corporation, Kyoto, Japan). The contents of flavonoids, saponins, and coumarins were subsequently determined using colorimetric assay methods with a UV-5500 UV–Vis spectrophotometer (Shanghai Metash Instruments Co., Ltd., Shanghai, China) at appropriate wavelengths. The standard phenolic compounds including gallic acid (Chemical Abstract Service (CAS) Registry Number: 149-91-7), catechin (CAS: 154-23-4), epicatechin (CAS: 490-46-0), naringin (CAS: 480-41-1) and phlorizin (CAS: 60-81-1) were purchased from Shanghai Standard Technology Co., Ltd. (Shanghai, China). The composition of polyphenolic compounds was determined using Shimadzu LC-40 HPLC (Shimadzu Corporation, Japan).

### 3.3. Quantitative and Qualitative Analyses

The quantitative and qualitative analyses of *A. glutinosa* leaves were performed by the methods described in the monographs [41,42]. This raw material analysis included their ash contents, moisture, extractive substances, organic acids, flavonoids, polysaccharides, alkaloids, coumarins, tannins and mineral composition. Their contents in the 96% ethanolic extract of *A. glutinosa* leaves were also assessed in accordance with the methods described in the State Pharmacopoeia of the Republic of Kazakhstan [43,44]

### 3.4. Mineral Composition Analysis

Powdered medicinal plant material (3 g) was placed in a pre-measured porcelain crucible, and the substance was evenly allocated across the bottom. The crucible was then gently heated on a hot plate. The remaining ash particles were burned at a low temperature; after the ash was almost completely burned, the temperature of flame increased. Calcination was continued until constant weight was reached at approximately 500 °C to ensure the ash melted and did not stick to the crucible walls. After calcination was complete, the crucible was cooled in a desiccator, and the resulting ash was then calcined again at 600 °C until it became uniformly gray. The resulting precipitate was dissolved in 5 mL of HNO_3_ (nitric acid) with heating. The resulting solution was heated on a hot plate until the salt became moist. The resulting solution was dissolved in 15 mL of 1 N HNO_3_ and transferred to a 25 mL volumetric flask, bringing the volume up to the mark. A parallel control experiment was conducted, in which a solution of the same concentration was prepared from the same acid in the same flask. The sample prepared according to the method described previously was analyzed by atomic absorption spectroscopy on an ASSIN instrument from Carl Zeiss (Jena, Germany). Spectra were measured using a DFS-13 (VMK-Optoelektronika, Novosibirsk, Russia) in the range of 2100–3600 Å; the sensitivity of the analysis was 10^−2^–10^−5^ [42].

### 3.5. GC-MS Analysis of Ethanolic Extract of A. glutinosa

The crude extract obtained by the extraction of 1 g of *A. glutinosa* leaves in 95% ethanol for 3 h was analyzed by gas chromatography–mass spectrometry (GC-MS) using mass spectrometric detection (7890A/5975C, Agilent Technologies, Santa Clara, CA, USA). Separation was performed using a DB-17ms chromatographic capillary column (Agilent Technologies, Santa Clara, CA, USA) with a length of 30 m, an internal diameter of 0.25 mm and a film thickness of 0.25 μm at a constant-carrier gas (helium) flow rate of 1 mL/min. The chromatography temperature was programmed from 40 °C with a heating rate of 5 °C/min to 280 °C (holding for 5 min). The analysis time was 63 min. Detection was carried out in the SCAN *m*/*z* 34-750 mode. The gas chromatographic system, registration and processing of the obtained results and data were controlled by the Agilent MSD ChemStation software (version 1701EA). To decipher the obtained mass spectra, the Wiley 7th edition and NIST’02 libraries were used [43,44].

### 3.6. Analysis of Polyphenolic Compounds Through HPLC

The presence of polyphenolic compounds in *A. glutinosa* leaves was determined using Shimadzu LC-40 high-performance liquid chromatography, including standard catechin, gallic acid, naringin, epicatechin and phlorizin. One milligram of dried 75% ethanolic extract of *A. glutinosa* leaves was dissolved in 1 mL of acetonitrile. The prepared 1 mL extract was filtered through a 0.22 μm syringe filter, the injection volume was 10 μL, the cell temperature was 40 °C, and the wavelength used was 272 nm. The identification of polyphenolic constituents was performed by the Eclipse XDB-C18 chromatographic column with a 4.6 mm × 250.0 mm and 5 μm (Agilent, Santa Clara, CA, USA) guard column at a flow rate of 1 mL/min. In the mobile phase, solvent B was acetonitrile, and solvent A was 0.1% of water–formic acid. A gradient elution was employed, beginning with 10% solvent B and linearly increasing to 90% over 55 min. The phenolic compounds were identified by comparing their retention time and peak areas with those of standards. All HPLC measurements were performed in triplicate, and the results are presented as mean ± standard deviation [41,42].

### 3.7. Antimicrobial Activity

The antimicrobial activity of the aqueous and fermented extract obtained from *A. glutinosa* leaves was evaluated. Fresh *A. glutinosa* leaves (pre-washed, air-dried, and ground into a coarse powder) were extracted with distilled water at a solid-to-liquid ratio of 1:10 (*w*/*v*) at 80 °C for 2 h with continuous stirring. The extract was then filtered and concentrated under reduced pressure to obtain an aqueous extract. The resulting extract was used to prepare various formulations. An alcohol tincture was prepared by mixing the aqueous extract with 96% ethanol in a 2:1 (*v*/*v*) ratio (Sample **1**). For fermentation, 8 L of the aqueous extract was mixed with 3% apple cider vinegar and 200 g of sucrose (Sample **2**). The mixture was maintained at 23 °C for 5–7 days. The fermented extract served as the basis for the preparation of formulations based on humic (Sample **3**) and fulvic acids (Sample **4**) with a final pH ≈ 3. To ensure reproducibility, all extracts were prepared in triplicate and stored at 4 °C until use. All prepared samples were subsequently tested for antimicrobial activity. These samples were diluted with distilled water at ratios of 1:1, 1:2, and 1:3 prior to testing. Antimicrobial activity was assessed against selected laboratory strains representing both Gram-positive and Gram-negative bacteria. The bacterial strains included *Staphylococcus aureus* ATCC 6538 (Gram-positive), *Salmonella abony* NTCC 6017 (Gram-negative), *Escherichia coli* NTCC 8439 (Gram-negative), and *Klebsiella pneumoniae* ATCC 700603 (Gram-negative). Antimicrobial activity was determined using the agar diffusion (well assay) method, and the bacterial suspension density was standardized using a 0.5 McFarland turbidity standard (~1 × 10^8^ CFU/mL), verified spectrophotometrically at 600 nm [13]. A standard microbial inoculum containing approximately 1.5 × 10^8^ CFU/mL was prepared from 24 h cultures. The suspension was added to 500 mL of melted meat–peptone agar (MPA) cooled to 60–65 °C, mixed thoroughly, and poured into Petri dishes (20 mL per plate). After solidification, the plates were dried for 30 min. Wells of 7 mm diameter were aseptically cut into the agar using a sterile drill, and 0.1 mL of each test solution was introduced into the wells. The plates were maintained at room temperature for 1 h to allow the diffusion of the extracts and then incubated in a thermostat. Bacterial cultures were incubated at 37 °C for 24 h and examined after 5 days. For quality control, solvent control groups (distilled water and 96% ethanol) were included in the antimicrobial assay to exclude any potential influence of the solvents on the observed activity. Gentamicin (10 µg/mL) was used as a positive control in the agar well diffusion assay to validate the sensitivity of the tested bacterial strains and to allow the comparative evaluation of antimicrobial activity.

### 3.8. Statistical Analysis

All experiments were performed in triplicate (*n* = 3), and the results are expressed as mean ± standard deviation (SD). Statistical analysis was carried out using the SigmaPlot software (version 10.0). Differences between groups were evaluated using one-way analysis of variance (ANOVA), followed by Tukey’s post hoc test. A *p*-value of less than 0.05 (*p* < 0.05) was considered statistically significant.

## 4. Conclusions

This study demonstrates that *A. glutinosa* is a rich source of biologically active compounds, supporting its long-standing use in traditional medicine. The physicochemical evaluation revealed a high level of extractive substances and tannins, indicating the suitability of the plant material for the isolation of bioactive constituents. The mineral analysis showed the predominance of essential macroelements, particularly K, Mg, and Ca, along with nutritionally important trace elements such as Fe, Zn, and Mn, while toxic heavy metals were absent or present only in negligible amounts, confirming the safety and quality of the raw material. GC-MS analysis identified 43 compounds in the ethanolic extract, with vitamin E, phytol, and squalene being the main components, indicating the predominance of lipophilic metabolites. HPLC analysis confirmed the presence of several phenolic and flavonoid compounds, with naringin being detected in the highest concentration among the quantified analytes. The results indicate that *A. glutinosa* leaves contain measurable amounts of both lipophilic and phenolic components, which may contribute to biological activity. Antimicrobial assays demonstrated moderate antibacterial activity against both Gram-positive and Gram-negative strains, and statistical analysis confirmed the significance of the observed differences between samples. The comparative evaluation of aqueous, alcoholic, and fermented preparations of *A. glutinosa* leaves suggests that composition and processing conditions may influence the observed antimicrobial effects. The antibacterial efficacy, particularly against *S. aureus* and *E. coli*, highlights the potential of *A. glutinosa* extracts as natural antimicrobial agents. Modifications using fermentation and acids enhanced the antimicrobial activity, with selective activity observed for certain formulations, particularly against *S. aureus* and *E. coli*. Overall, the findings provide a scientific basis for the ethnomedicinal applications of *A. glutinosa* and emphasize its potential as a valuable natural source of bioactive compounds for pharmaceutical, nutraceutical, and preventive health applications. Further studies focusing on biological mechanisms, toxicity assessments, and in vivo efficacy are recommended to support its future therapeutic development.

## Figures and Tables

**Table 1:** Quantitative and qualitative analyses of bioactive ingredients of *A. glutinosa* leaves.

No	Composition of *A. glutinosa* Leaves	Content, %
1	Ash contents	6.740 ± 0.023
2	Moisture	5.000 ± 0.012
3	Polysaccharides	0.421 ± 0.004
4	Tannins	7.439 ± 0.011
5	Alkaloids	0.205 ± 0.002
6	Coumarins	0.257 ± 0.002
7	Extractive substances	17.684 ± 0.245
8	Organic acids	0.388 ± 0.005
9	Flavonoids	0.322 ± 0.003

All measurements were performed in triplicate (*n* = 3), and the results are presented as mean ± standard deviation (SD). Statistical analysis was performed using SigmaPlot 10.0.

**Table 2:** Mineral сomposition of macro- and microelements in the ash of *A. glutinosa* leaves.

No	Elements	Conc. in Ash, mg/g
1	Cu	0.4121 ± 0.002
2	Fe	1.2266 ± 0.011
3	Zn	0.8870 ± 0.012
4	Ni	0.2167 ± 0.005
5	Mn	0.8141 ± 0.002
6	Pb	-
7	Cd	0.0170 ± 0.004
8	Ca	75.2989 ± 0.605
9	Mg	97.1287 ± 0.453
10	K	11.4330 ± 0.152
11	Na	72.4091 ± 0.212

All measurements were performed in triplicate (*n* = 3), and the results are presented as mean ± standard deviation (SD). Statistical analysis was performed using SigmaPlot 10.0.

**Table 3:** Main compounds of *A. glutinosa* leaves identified by GC-MS.

No	RT	Compound Name	MF	MW (g/mol)	Content (%)
**1**	13.59	2-Hydroxy-gamma-butyrolactone	C_4_H_6_O_3_	102.09	1.68
**2**	13.99	3H-Pyrazol-3-one, 2,4-dihydro-2,4,5-trimethyl-	C_6_H_10_N_2_O	126.16	1.04
**3**	15.96	4*H*-Pyran-4-one, 2,3-dihydro-3,5-dihydroxy-6-methyl-	C_6_H_8_O_4_	144.13	1.93
**4**	16.71	Cyclopropyl carbinol	C_4_H_8_O	72.11	1.25
**5**	20.24	5-Hydroxymethylfurfural	C_6_H_6_O_3_	126.11	4.19
**6**	20.69	2-Methoxy-4-vinylphenol	C_9_H_10_O_2_	150.17	1.06
**7**	27.19	Sucrose	C_12_H_22_O_11_	342.30	5.42
**8**	27.99	3′,5′-Dimethoxyacetophenone	C_10_H_12_O_3_	180.20	1.33
**9**	28.29	Megastigmatrienone	C_13_H_18_O	190.28	1.89
**10**	29.24	β-D-Glucopyranose, 4-*O*-β-D-galactopyranosyl-	C_12_H_22_O_11_	342.30	1.04
**11**	31.27	3-Deoxy-d-mannoic lactone	C_6_H_10_O_5_	162.14	1.31
**12**	32.33	Hexadecanoic acid	C_16_H_32_O_2_	256.43	7.04
**13**	32.71	4-((1*E*)-3-Hydroxy-1-propenyl)-2-methoxyphenol	C_10_H_12_O_3_	180.20	1.61
**14**	34.53	Phytol	C_20_H_40_O	296.53	12.46
**15**	36.81	9,12,15-Octadecatrienoic acid, (*Z*,*Z*,*Z*)-	C_18_H_30_O_2_	278.43	2.89
**16**	46.08	Squalene	C_30_H_50_	410.73	10.29
**17**	49.24	Eicosanoic acid, 2-hydroxy-1-(hydroxymethyl)ethyl ester	C_23_H_46_O_4_	386.60	1.31
**18**	51.64	γ-Tocopherol	C_28_H_48_O_2_	416.68	1.79
**19**	53.40	Vitamin E	C_29_H_50_O_2_	430.71	20.52
**20**	59.70	γ-Sitosterol	C_29_H_50_O	414.71	7.48
**21**	62.35	13,27-Cycloursan-3-one	C_30_H_48_O	424.70	1.27
**22**	62.64	Olean-12-en-3-one	C_30_H_48_O	424.70	1.29

**Table 4:** The results of the chromatographic analysis of the standard compounds present in *A. glutinosa*.

Retention Time (min)	Square	Height	Concentration (mg/L)	R^2^	Name
4.435	206,068	14,049	65.031 ± 0.121	0.9911	Gallic acid (**1**)
8.373	63,691	4214	64.828 ± 0.981	0.9987	Catechin (**2**)
10.443	71,059	5162	64.272 ± 1.105	0.9990	Epicatechin (**3**)
17.422	132,983	11,244	34.313 ± 0.762	0.9991	Naringin (**4**)
19.612	149,135	12,075	64.180 ± 1.213	0.9985	Phloridzin (**5**)

Values are expressed as mean ± SD (*n* = 3). Calibration curves were constructed using external standards (R^2^ ≥ 0.998). Method precision and repeatability were within acceptable limits.

**Table 5:** Results of the quantitative analysis of standard compounds in the *A. glutinosa* extract.

Retention Time (min)	Square	Height	Concentration (mg/L)	R^2^	Name
4498	1274	248	0.402 ± 0.044	0.9911	Gallic acid (**1**)
8478	12,266	966	12.485 ± 0.435	0.9987	Catechin (**2**)
10,323	16,719	913	15.123 ± 0.765	0.9990	Epicatechin (**3**)
17,188	116,665	7831	56.421 ± 1.523	0.9991	Naringin (**4**)
19,601	2763	268	11.800 ± 0.921	0.9985	Phloridzin (**5**)

Values are expressed as mean ± SD (*n* = 3). Calibration curves were constructed using external standards (R^2^ ≥ 0.998). Method precision and repeatability were within acceptable limits.

**Table 6:** Activity of aqueous and fermented *A. glutinosa* extracts and their dilutions.

Sample No	Diameter of the Zone of Inhibition of Growth of the Studied Microorganisms, mm				
	Dilution	*S. aureus* 6538	*S. abony* NTCC 6017	*E. coli* NTCC 8439	*K. pneumonia* 700603
**1**	Initial	21.5 ± 0.3	10.8 ± 0.1	10.5 ± 0.4	10.6 ± 0.1
	1:1	16.7 ± 0.2	0	0	0
	1:2	11.5 ± 0.4	0	0	0
	1:3	0	0	0	0
**2**	Initial	18.7 ± 0.1	17.6 ± 0.2	16.2 ± 0.4	16.5 ± 0.3
	1:1	12.5 ± 0.3	12.8 ± 0.4	10.9 ± 0.1	10.5 ± 0.2
	1:2	0	0	0	0
	1:3	0	0	0	0
**3**	Initial	18.6 ± 0.1	18.5 ± 0.3	20.8 ± 0.1	15.4 ± 0.2
	1:1	12.3 ± 0.2	10.3 ± 0.1	10.8 ± 0.3	10.6 ± 0.4
	1:2	0	0	0	0
	1:3	0	0	0	0
**4**	Initial	18.2 ± 0.4	16.3 ± 0.2	17.8 ± 0.1	15.3 ± 0.3
	1:1	12.0 (bacterio-static) ± 0.3	12.6 ± 0.1	12.0 (bacterio-static) ± 0.2	10.0 (bacterio-static) ± 0.1
	1:2	0	0	0	0
	1:3	0	0	0	0
Gentamicin (10 µg/mL)	-	24.3 ± 0.2	22.8 ± 0.3	25.1 ± 0.1	23.6 ± 0.2

## Data Availability

The original contributions presented in this study are included in the article. Further inquiries can be directed to the corresponding authors.
